# Identification of *Escherichia coli* from broiler chickens in Jordan, their antimicrobial resistance, gene characterization and the associated risk factors

**DOI:** 10.1186/s12917-019-1901-1

**Published:** 2019-05-22

**Authors:** Rekaz A. Ibrahim, Tillie L. Cryer, Shawkat Q. Lafi, Ehab-Abu Basha, Liam Good, Yaser H. Tarazi

**Affiliations:** 1 Department of Basic Medical Veterinary Sciences, Jordan University of Science and Technology, Irbid, Jordan; 2 Department of Pathology and Public Health, Jordan University of Science and Technology, Irbid, Jordan; 3 Pathobiology and Population Sciences Department, the Royal Veterinary College, London, UK

**Keywords:** APEC, Antimicrobial Resistance, Broiler chickens, Colibacillosis, Risk factors, Jordan

## Abstract

**Background:**

Avian pathogenic *Escherichia coli* (APEC) is the principle cause of colibacillosis affecting poultry. The main challenge to the poultry industry is antimicrobial resistance and the emergence of multidrug resistant bacteria that threaten the safety of the food chain. Risk factors associated with emergence of antimicrobial resistance among avian pathogenic *E. coli* were correlated with the inappropriate use of antimicrobials along with inadequate hygienic practices, which encourages the selection pressure of antimicrobial resistant APEC. The aim of this study was to isolate, identify, serogroup and genotype APEC from broilers, assess their antibiotic resistance profile, expressed genes and the associated risk factors.

**Results:**

APEC was isolated from the visceral organs of sick chickens with a prevalence of 53.4%. The most prevalent serotypes were O1, O2, O25 and O78, in percentage of 14.8, 12.6, 4.4 and 23.7%, respectively. Virulence Associated Genes; SitA, iss, iucD, iucC, astA, tsh cvi and irp2 were detected in rate of 97.4, 93.3, 75, 74, 71, 46.5, 39 and 34%, respectively and 186 (69.2%) isolates possess > 5–10 genes. The highest resistance was found against sulphamethoxazole-trimethoprim, florfenicol, amoxicillin, doxycycline and spectinomycin in percentage; 95.5, 93.7, 93.3, 92.2 and 92.2%, respectively. Sixty-eight percent of APEC isolates were found to have at least 5 out of 8 antimicrobial resistant genes. The most predominant genes were Int1 97%, tetA 78.4%, bla TEM 72.9%, Sul1 72.4%, Sul2 70.2%. Two risk factors were found to be associated with the presence of multi-drug resistant APEC in broiler chickens, with a *P* value ≤0.05; the use of ground water as source of drinking water and farms located in proximity to other farms.

**Conclusions:**

This study characterized the VAGs of avian pathogenic *E. coli* and establish their antimicrobial resistance patterns. The widespread of antimicrobial resistance of APEC isolates and detection of ARGs highlighted the need to monitor the spread of ARGs in poultry farms and the environment in Jordan. Use of ground water and closely located farms were significant risk factors associated with the presence of MDR APEC in broiler chickens in Jordan.

**Electronic supplementary material:**

The online version of this article (10.1186/s12917-019-1901-1) contains supplementary material, which is available to authorized users.

## Background

Avian pathogenic *E. coli* causes localized or systemic infection outside the avian gut, which indicates as Extraintestinal Pathogenic *E. coli* (ExPEC). The infection caused by ExPEC is termed colibacillosis which is an infectious disease characterized by acute fatal septicemia or sub- acute fibrinous pericarditis, airsacculitis, salpingitis, and peritonitis affect broiler chickens aged 4–6 weeks [1, 2]. Colibacillosis is a common bacterial disease of economic importance in poultry through decreasing the infected birds’ productivity, increase mortality, condemnation of infected carcasses at slaughter, and prophylaxis and treatment cost [2] and is reported worldwide.

APEC is considered a primary or secondary pathogen of poultry. Strains which carry virulence genes (adhesin, invasins, toxins, resistance to host serum, iron acquisition systems, temperature-sensitive hemagglutinin, and K1 capsule) have all been shown to contribute to APEC pathogenesis [3, 4] and could induce colibacillosis without previous immune suppression factors; stress or concurrent infections [5].

The control and prevention of bacterial diseases in food animals is achieved by the application of antimicrobials during the periods of high risk of infectious bacterial diseases, as prophylactic treatment, and as growth promoters [6].

Bacterial antimicrobial resistance develops naturally over time; the unprecedented increase of antimicrobial resistant organisms is linked to the massive use of antimicrobial agents for disease control and prevention in human and animal medicine [7]. Several forces play a role in the spread of antimicrobial resistant bacteria include the presence of carrier animal moving between animal herds and through vector action [8].

The key points in controlling avian colibacillosis are management interventions, infections control and vaccination strategies [2]. Wide range of antimicrobial agents is used in poultry colibacillosis treatment, which include: β-lactams (penicillins, cephalosporin), aminoglycosides, tetracycline, sulphonamides and fluoroquinolones [9]. The frequent use of antimicrobial agents give rise to selective pressure that lead to antimicrobial resistance against APEC [10].

The development of resistance is a complex process associated with the presence of resistance encoding genes that are found inside plasmids or chromosomal genetic material. Integrons are the genetic material responsible for capturing resistance genes that spread via the genetic mobile elements; transposons and plasmid. The presence of integrons is detected by amplification of integrase genes (intI 1, intI2 and intI 3) [11]. Resistance to tetracycline is mediated through efflux pump system which encoded by tetracycline resistance group of genes (tetA, tetB, tetC, tetD, tetE and tetG) [12]. Phenicols resistance encoding genes are (cat1, cat2, cat3, cmlA and cmlB) [13] aminoglycosides resistance genes are (strA, strB, addA1, addA 2) [14] and genes responsible for sulphonamide resistance are (sul 1, sul 2 and sul 3) [15].

Antimicrobial resistant *E. coli* strains pose a serious problem for public health, since these strains could be passed to humans via the food chain or by direct contact with infected birds. In addition, resistant *E. coli* may act as transporters for antimicrobial resistant genes to other pathogens [16].

In many developed countries, administration of antimicrobial agents is not only restricted for treatment purpose. Antimicrobials can also be used to enhance animal productivity, feed conversion rate and growth rate in food producing animals [17]. This type of farming practice allows antimicrobial drugs to eliminate sensitive bacterial strains and select strains with genetic traits that can resist antimicrobials, which provides favourable conditions for selected strain persistence and spread at the farm level [18].

The use of antimicrobial agents as feed additives, administered at low concentrations (sub-therapeutic dose) usually over long periods of time, may lead to development of resistance [19, 20]. Other risk factors include: the breed of the animal, dose, duration of treatment, capacity of the farm, and animal husbandry practices [21]. Poor hygiene and lack of commitment with control measures and disease prevention have participated in the propagation and expansion of antimicrobial resistant strains [22].

Resistant bacteria could be shed in the faeces and passed into sewage systems, which are considered as suitable transporters for resistance genes and the spread of resistant bacteria into the wider environment. Antibiotic residues and by-products found in municipal sewage, waste water treatment plants, and soil, are flushed into rivers by surface water and reach ground water resources [23].

The use of disinfectants to limit infection transmission between animals subsequently increases animal health and productivity. Quaternary ammonium compounds (QACs) may have the potential to induce the emergence of antimicrobial resistance, which could be raised from cross-resistance between QACs and a range of antimicrobials [24, 25]. The use of chicken litter-based organic fertilizers in the presence of antimicrobial resistance pathogens are considered as a serious environmental hazard, as the spread of fertilizers on pasture could contaminate ground water sources and land that may facilitate the transmission of antimicrobial resistant pathogens to other animal species and humans. This highlights that proper waste management could be effective in controlling the spread of antimicrobial resistance pathogens [21, 26]. Antimicrobial resistance has also been reported in wildlife, indicating that the common habitat between wildlife, food animals, water sources and environmental contamination has resulted in the transmission of antimicrobial resistant bacterial pathogens into the food chain as well as their role in contaminating foods of plant origin [27].

Therefore, the objectives of the current study are to isolate and identify *E. coli* from live sick birds, establish their serotypes, their virulence associated genes, antibiotic resistance profiles and their associated genes and to identify risk factors and farming practice associated with the antimicrobial resistance *E. coli*.

## Results

### *E. coli* isolation

A total of 504 broiler chicken samples (from 84 broilers farm) were cultured, 269 (53.4%) isolates were confirmed as *E. coli* by conventional and RapID™ ONE System and were used for further molecular and antimicrobial testing.

### Molecular identification of *E. coli* by PCR

**Fig. 1:**
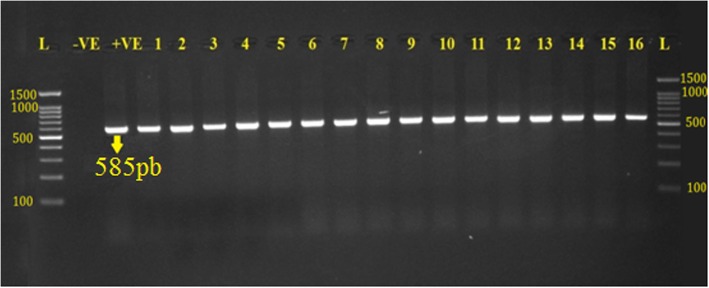
Products of PCR for the detection of 16 s rRNA gene on 1.5% EB-stained agarose gel amplified from APEC isolates from broilers, where L 100 bp DNA ladder; −ve is negative control; +ve is positive control *E. coli* ATCC 25922; lane 1–16: *E. coli* isolates

All isolates that were confirmed as *E. coli* by the RapID™ ONE system also underwent PCR to further confirm the isolates as *E. coli.* The universal primer for 16 s RNA with 585 bp band size was used. *Escherichia coli* ATCC 25922 was used as positive control as demonstrated by (Fig. 1).

### APEC serotyping

All confirmed *E. coli* isolates were serotyped. One hundred eighty-nine (70.3%) were identified as eleven different serotypes using the available antisera; O1, O2, O9, O18, O25, O26, O78, O111, O114, O119, O127. Whereas, the remaining isolates; 54 (20%) were untypeable and 26 (9.66%) were rough strains that show autoagglutination, serotypes and their frequencies are shown in (Table 1).

**Table 1:** Distribution of *E. coli* serotypes isolated from broiler chicken farms in north Jordan

O-serotypes	No. of isolates (percent %)	Geographical distribution of the serotypes			
		Irbid	Jarash	Mafraq	Ajlune
O1	40 (14. 9)	14	9	7	10
O2	34 (12.6)	13	6	7	8
O9	8 (3.00)	3	2	1	2
O18	4 (1.5)	2	0	2	0
O25	12 (4.5)	5	2	3	2
O26	2 (0.7)	1	0	1	0
O78	64 (23.8)	23	16	11	14
O111	3 (1.00)	0	1	1	1
O114	9 (3.3)	3	3	0	3
O119	11 (4)	4	2	4	1
O127	2 (0.7)	1	0	1	0
Untypeable	54 (20)	18	17	2	17
Rough	26 (9.7)	8	7	5	6
Total	269 (100)	95	65	45	64

### Multiplex polymerase chain reaction method for detection of virulence associated genes (VAGs)

**Fig. 2:**
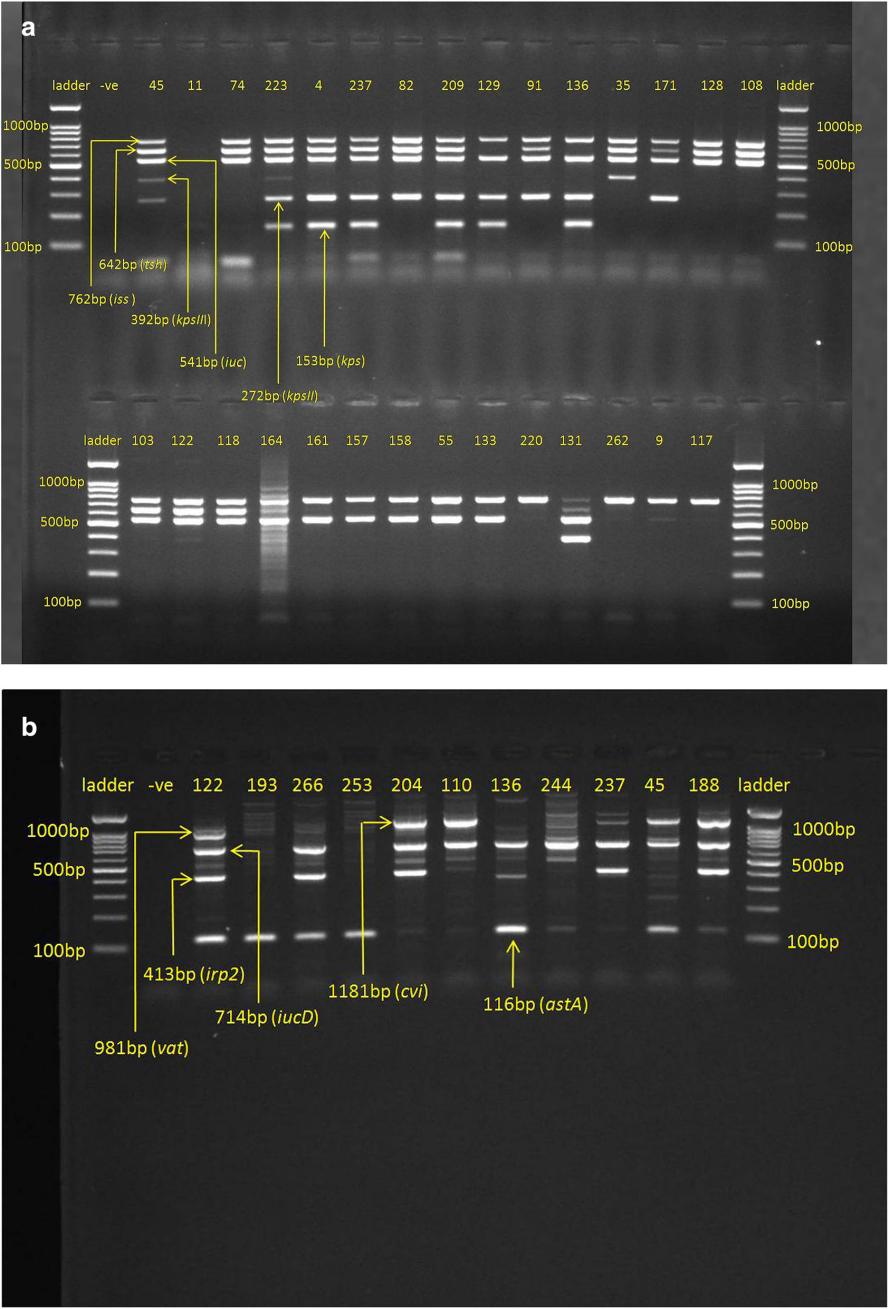
**a** PCR Products for detection of virulence genes tsh gene 642 bp, iss gene 762 bp, kpsIII gene 392 bp, kpsII gene 272 bp, iuc gene 541 bp, ksp gene 153 bp. **b** PCR Products for detection of virulence genes vat gene 981 bp, iucD gene 714 bp, irp2 gene 413 bp, cvi gene 1181 bp, astA gene 116 bp

Sixteen virulence associated genes were investigated using multiplex PCR, for avian *E. coli* indicates that *sitA* is the most prevalent gene (262, 97.4%) followed by iss (251, 93.3%), iucC (199, 74%), iucD (203, 75%), astA (190, 71%), tsh (125, 46.5%), cvi (106, 39%), irp2 (91, 34%), KpsII (33, 12.3%), KPS (20, 7.4%), KpsIII (13, 4.8%) and vat (7, 2.6%). *HlyD* and *ibeA* were not detected and *papC* and *sfa* were detected in one isolate each among the 269 *E. coli* tested (Fig. 2a, b).

One hundred eighty-six (69.2%) of the 269 *E. coli* tested isolates possess > 5–10 VAGs. In detail; 3 isolates possessed 10 VAGs, 17 isolates revealed 9 genes, 38 isolates revealed 8 genes, 60 isolates revealed 7 genes, 40 isolates revealed 6 genes, 28 isolates revealed 5 genes, 25 isolates revealed 4 genes, 55 isolates revealed 3 genes, 2 isolates revealed 2 genes, 4 isolates revealed one gene and 4 isolates revealed no genes.

### Antibiotic susceptibility test

#### Standard disc diffusion method

The highest levels of antimicrobial resistance were found against sulphamethoxazole-trimethoprim, florfenicol, amoxicillin, doxycycline and spectinomycin in percentage of; 95.5, 93.7, 93.3, 92.2 and 92.2%, respectively (Table 2).

**Table 2:** Frequency of antimicrobial resistance among APEC isolates from broiler chickens by disc diffusion method

Antimicrobials (abbreviation)	Disc content (μg)	Disc diffusion interpretive criteria^a^ (mm)		*E. coli* (*n* = 269)
		R	S	Number ( % )of resistant isolates
β – lactams				
Amoxicillin (AX)	25	<14	≥14	251 (93.3%)
Azetronem (ATM)	30	≤21	≥26	15 (5.6%)
Imipenem (IPM)	10	≤16	≥22	0 (0%)
β – lactamase inhibitors				
amoxicillin – clavulanic acid (AML)	20/10	<19	≥19	190 (70.6%)
Tetracyclines				
Doxycycline (DO)	30	≤10	≥ 14	248 (92.2%)
Oxytetracyclin (OT)	30	≤11	≥15	148 (55%)
Chlortetracycline (CHL)	10	≤13	≥17	201 (74.7%)
Sulfonamides				
Sulphamethoxazole-trimethoprim (SXT)	23.75/1.25	≤11	≥14	257 (95.5%)
Fluoroquinolones				
Enrofloxacin (ENR)	5	≤15	≥21	227 (84.4%)
Ciprofloxacin (CIP)	5	≤24	≥26	172 (63.9%)
Aminoglycosides				
Spectinomycin (SH)	25	≤11	≥15	248 (92.2%)
Gentamicin (CN)	10	≤14	≥17	154 (57.2%)
Apramycin (APR)	15	≤12	≥15	147 (54.6%)
Cephalosporin				
Cephalexin (CL)	30	<14	≥14	236 (87.7%)
Ceftazidime (CZC)	30	≤19	≥22	20 (7.4%)
Ceftriaxone (CRO)	30	≤22	≥25	13 (4.8%)
Cefepime (FEP)	30	≤24	≥27	9 (3.3%)
Phosphoric acid derivatives				
Fosfomycin (FOS)	50	<24	≥24	80 (30%)
Phenicol				
Florfenicol (FFC)	30	≤10	≥21	252 (93.7%)

^a^Interpretive criteria: depends on reference strain *E. coli* ATCC 25922, demonstrated in CLSI 2012, supplement M100-S22, Vol.32, No.3, Table 2A

#### Minimal inhibitory concentration (MIC)

MIC was performed on all APEC isolates using eight different antimicrobial agents based on their common use in poultry sector. Results illustrated in Table 3 were interpreted according to animal criteria by clinical and laboratory standard institute [28]. *Escherichia coli* ATCC 25922 was used as control for each run of the test.

**Table 3:** Minimal inhibitory concentration test results for 269 APEC isolates, CLSI (2012)

Antimicrobials Agents	Number & (%) of APEC isolates			MIC^a^ interpretive criteria^c^		
	R^b^	I	S	R	I	S
Ceftriaxone	15 (5.5)	8 (2.97)	245 (91)	≥4	2	≤1
Ceftazidime	21 (7.8)	5 (1.85)	243 (90.3)	≥16	8	≤4
Gentamicin	160 (59.4)	42 (15.6)	67 (24.9)	≥16	8	≤4
Ciprofloxacin	178 (66)	11 (4)	80 (29.7)	≥4	2	≤1
Cephalexin	238 (88.4)	26 (9.66)	5 (1.85)	≤16	8	≥3
Doxycycline	251 (93.3)	3 (1.1)	15 (5.57)	≤16	8	≥4
Amoxicillin	254 (94.4)	15 (5.57)	0 (0)	≤32	16	≥8
Florfenicol	258 (95.9)	11 (4)	0 (0)	≤8	4	≥2

^a^ MIC: minimal inhibitory concentration of *E. coli* ATCC 25922, ^b^ R: resistant, I: intermediate resistance, S: sensitive. ^c^ The MIC interpretive criteria of *E. coli* ATCC 25922 for Ceftriaxone, Ceftazidime, Gentamicin and Doxycycline is the same value as of breakpoint published by CLSI document M100-S26. CLSI 2017, M100, 27th ed., for ciprofloxacin. For amoxicillin and cephalexin according to EUCAST Clinical Breakpoint Tables v. 8.1, valid from 2018 to 05-15, for Florfenicol according to NCCLS document M7-A3, 1999

#### Detection of antimicrobial resistant genes by multiplex PCR

**Fig. 3:**
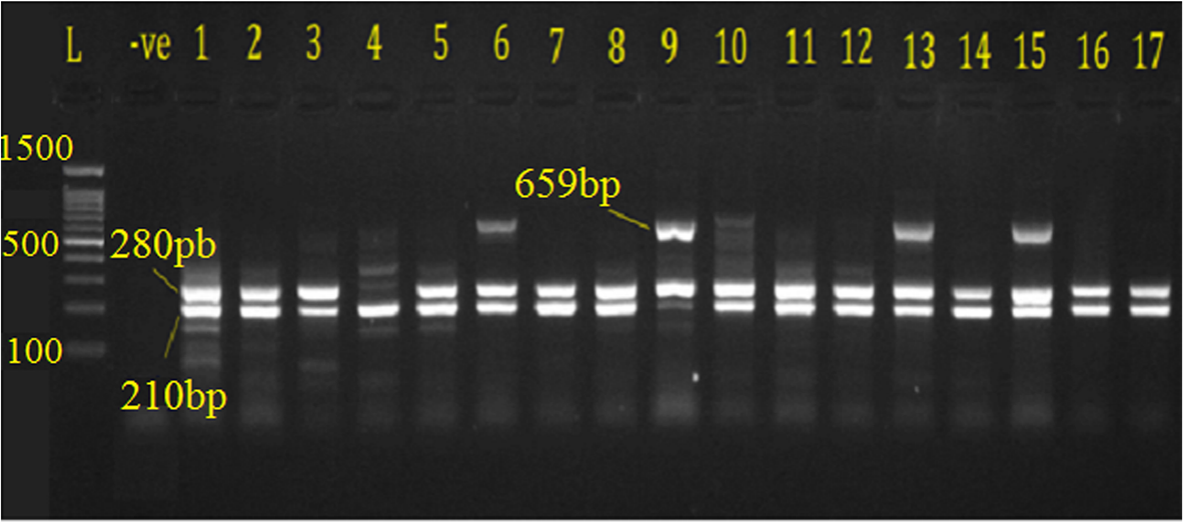
PCR products for detection of TetA gene 210 bp, TetB gene 659 bp and Int1 gene 280 bp on 1.5% EB-stained agarose gel amplified from APEC isolated from broilers, where L 100 bp DNA ladder; −ve is negative control; 1–17 lanes; E. coli isolates

**Fig. 4:**
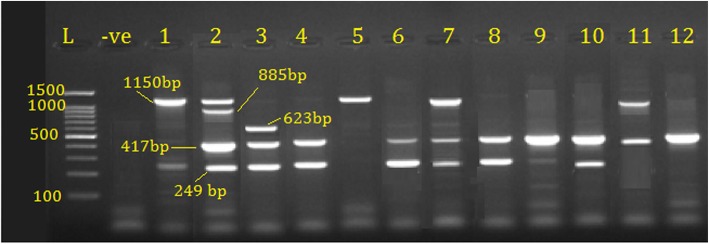
PCR products for detection of sul2 gene 249 bp, sul1 gene 417 bp, cat gene 623 bp, bla SHV gene 885 bp and bla TEM 1150 bp, on 1.5% EB-stained agarose gel amplified from APEC isolated from broilers, where L 100 bp DNA ladder; −ve is negative control; 1–12 lanes; E. coli isolates

DNA’s templates from the extraction step were used to detect the prevalence of eight antimicrobial resistance genes (ARG) among APEC isolates by multiplex PCR (Table 4). The eight antimicrobial resistance genes were present in different combinations, ranging from two genes in some isolates to eight genes in others. All isolates had at least two ARGs, 183(68%) of *E. coli* isolates found to possess at least 5 out of 8 ARGs, while only 3(1.1%) were found to have all the eight tested genes (Figs. 3 and 4 ).

**Table 4:** Prevalence of antimicrobial resistance genes in 269 APEC isolates from broiler chickens in Jordan

	Antimicrobial resistance genes							
	*tetA*	*tetB*	*int 1*	*sul 1*	*sul 2*	*shv*	*tem*	*Cat*
Prevalence (%)	211 (78.4)	82 (30.5)	261 (97)	195 (72.4)	190 (70.6)	5 (1.8)	199 (72.9)	166 (61.7)

### Risk factors analysis

#### Data analysis

After excluding variables with the same answers, chi-square (X^2^) and fisher exact tests were performed to screen association between outcome variable (resistance status of the farm) and risk factors in univariable analysis. Twenty-nine variables included in the univariable analysis screening, only variables with P ≤ 0.25 considered for further analysis (Table 5). Nine variables have *P* value ≤0.25 used to perform the final logistic regression model. Collinearity between variables was tested using chi-square and spearman rank correlation test in bivariate analysis. Results of (X^2^) are shown in (Table 5) and results of spearman rank correlation test (Additional file 2).

**Table 5:** Potential risk factors for the presence of multidrug resistant APEC isolates in broiler farms

Variables (risk factors)	Coding	Description		Resistance status		X^2^ *p*-value***
		No*	%	R**	S	
Geographical location of the farm						
Plain	0	16	19	9	7	0.977
Mountain	1	53	63.1	31	22	
Desert	2	15	17.9	9	6	
Poultry house direction^a^						
South to north	0	57	67.9	22	35	0.00
East to west	1	27	32.1	27	0	
Distance from the high way and noise^a^						
On the high way	0	40	47.6	31	9	0.001
Far from the high way	1	44	52.4	18	26	
Type of ventilation system						
Natural	0	83	98.8	49	34	0.234^b^
Mechanical	1	1	1.2	0	1	.417
Number of houses in the farm						
One	0	36	42.9	21	15	1.0
Multiple	1	48	57.1	28	20	
Distance in relation to other poultry farms^a^						
Isolated	0	32	38.1	7	25	0.00
Very close	1	52	61.9	42	10	
Presence of wild birds and rodents in the farm						
No	0	1	1.2	0	1	0.234^b^
Yes	1	83	98.8	49	34	.417
Application of pest control program						
No	0	12	14.3	8	4	0.527
Yes	1	72	85.7	41	31	
Frequency of litre discard						
Daily	0	29	34.5	19	10	0.332
Weekly	1	55	65.5	30	25	
Presentation of feed material						
Grounded	0	7	8.7	2	5	0.095^b^
Pellets	1	77	91.7	47	30	0.122
Water source^a^						
Municipalities	0	64	76.2	31	33	0.001
Artesian wells	1	20	23.8	18	2	
Water tanks type						
Cement	0	10	11.9	6	4	0.909^b^
Metallic	1	74	88.1	43	31	1.000
Frequency of water tanks cleaning						
Monthly	0	9	10.7	4	5	0.011^b^
When needed	1	15	17.9	13	2	
Between cycles	2	55	65.5	27	28	
Weekly	3	5	6	5	0	
Frequency of water tanks disinfecting						
Monthly	0	9	10.7	4	5	0.007^b^
When needed	1	19	22.6	16	3	
Between cycles	2	51	60.7	24	27	
Weekly	3	5	6	5	0	
Type of disinfectants used						
One type	0	58	69	35	23	0.576
Mixed types	1	26	31	14	12	
Disinfect farm entrance for visitors and vehicles						
No	0	49	58.3	26	23	0.246
Yes	1	35	41.7	23	12	
Workers wear protective cloth when handling birds^a^						
No	0	56	77.4	49	16	0.00
Yes	1	19	22.6	0	19	
Restricted entrance against unauthorized traffic^a^						
No	0	13	15.5	12	1	0.006
Yes	1	71	84.5	37	34	
Number of workers in the farm						
One	0	66	78.6	38	28	0.787
More than one	1	18	21.4	11	7	
Use antibiotic for disease prevention^a^						
No	0	51	60.7	26	25	0.089
Yes	1	33	39.3	23	10	
Use antibiotic as growth promotion^a^						
No	0	67	79.8	36	31	0.089
Yes	1	17	20.2	13	4	
Get prescription before use antimicrobials^a^						
No	0	8	9.5	7	1	0.079^b^
Yes	1	76	90.5	42	34	0.131
Perform necropsy before prescribing antibiotics						
No	0	34	40.5	21	13	0.599
Yes	1	50	59.5	28	22	
Information source about antibiotics^a^						
Veterinarian	0	47	56	24	23	0.021
Drug store	1	5	6	1	4	
Other (neighbours,training)	2	32	38.1	24	8	
Keep antibiotics in the farm						
No	0	2	2.4	0	2	0.090^b^
Yes	1	82	97.6	49	33	0.171
Perform antibiotic sensitivity before treatment						
No	0	47	56	28	19	0.795
Yes	1	37	44	21	16	
Frequency of antibiotics use during the cycle (~ 6 weeks)						
Less than five times	0	24	28.6	15	9	0.624
Five or more times	1	60	71.4	34	26	
Frequency of vet visits to the farm						
Once weekly	0	46	54.8	28	18	0.170^b^
When needed	1	31	36.9	15	16	
Never	2	7	8.3	6	1	

* No: number of farms, **R: resistant farms (*n* = 49), S: sensitive farms (*n* = 35), ***X^2^ *p*- value: chi- square value for potential risk factors and resistant status of the farm, ^a^: statistically significant at *P* ≤ 0.25 (two-sided), ^b^: fisher exact test was performed instead of X^2^ when variables had expected count less than 5 in one or more cells

#### Independent variable

The resistance status of each individual farm was used as unit of comparison, out of 84 farms completed the questionnaire; 49 (58.3%) resistance farm (presence of one or more multidrug resistant APEC isolate) coded as (1) Multidrug resistance is defined as a single bacterial isolate resistant to 3 or more antimicrobial classes (43), and 35 (41.7%) susceptible farms (no MDR-APEC present in the farm) coded as (0).

#### Final multivariable logistic regression

Nine variables from univariate analysis step were used to perform multivariable logistic regression model for the outcome, risk factors were considered significant when *P* value ≤0.05, non-significant factors re-entered when a new variable become significant or removed. Two variables with *P*-value ≤0.05 (water source and distance in relation to other farms) and two variables approaching significance with *P*-value ≤0.10 (use of antimicrobials as growth promoters and get prescription before antimicrobial treatment) remain in the final model (Table 6). The final model was tested to fit Hosmer and Lemeshow-of-fit test [29].

**Table 6:** Final logistic regression model for risk factors associated with multidrug resistance APEC isolates

Variables	b	S.E.	wald	Df	Sig. *p*-value	OR	95% C.I. for OR	
							Lower	Upper
Use of antimicrobials as growth promoters								
No	1.492	.854	3.053	1	.081	4.446	.834	23.700
Yes								
Water source								
Municipalities	2.895	1.019	8.067	1	.005	18.090	2.453	133.400
Artesian wells								
Distance in relation to other farms								
Isolated	3.169	.735	18.608	1	.000	23.774	5.635	100.312
Very close								
Get prescription before antibiotic treatment								
No	2.599	1.482	3.073	1	.080	13.448	.736	245.780
Yes								
Constant	−2.492-	.688	13.136	1	.000	.083		

Hosmer and Lameshow test X^2^ 3.31 sig .507

## Discussion

### *E. coli* isolation

Colibacillosis is caused by APEC, which considered as one of the major threat to poultry industry and public health. In present study*,* APEC was isolated from broiler chickens in northern Jordan, with a clinical manifestation of colibacillosis at a prevalence rate of 53.4%. In Jordan, two investigations of broiler chickens with colibacillosis have been previously carried out with prevalence rate of 88.2 and 77% [30, 31], respectively. In other countries, the prevalence rates of colibacillosis range from 52.26 to 86.7% [32–35].

The high prevalence of *E. coli* infections in broiler chickens could be associated with the accumulation of *E. coli* aerosols in the atmosphere of chicken barns that are inhaled by chickens into the respiratory tract. Samples that gave negative bacterial culture may be collected from farms that used early antibiotic treatment policy. *E. coli* isolation was from chicken visceral organs which are the last stage of the disease colonization [36]. RapID one system conformation and molecular identification were performed to reduce the false positive results.

### *E. coli* serotypes

In the current study, serotypes O78, O1, and O2 were identified at a prevalence of 23.79, 14.86, and 12.63%, respectively. In Jordan, a study by Al-Tarazi [31] demonstrated that prevalent serotypes were O78 (8%), O1a and O1b (5.2%), O8 (4.8%), O127aO127b (4.8%), and O45 (4.5%) which was isolated from cases of broiler colibacillosis and egg peritonitis. However, similar results to our findings were presented in Egypt and Iran [37, 38]. In China and Northern Ireland, O78 was found as a predominant serotype in cases of broiler colibacillosis [39, 40]. It is clear that the results from this study and other previous evidence that O78, O2 and O1 were the most prevalent APEC serotypes in broiler chickens. Table 1 indicates that all serotypes are present in the four governorates included in this study which highlight that is no control measures to prevent spreading of the APEC.

Serotype O18 was identified in 1.5% of *E. coli* isolates that share common phenotypic and genotypic characteristics with human ExPEC and NMEC strains. This may explain the zoonotic potential of those strains [41]. Other serotypes were isolated in less frequency, and they are of less important for poultry industry.

### Virulence associated genes (VAGs)

Screening multiplex PCR for sixteen VAGs was performed for all isolates; the most prevalent genes were SitA (97.4%), iss (93.3%), iucC & D (75%), astA (71%), tsh (46.5%) and cvi (39%) genes. Presence of three out of four of iss, iucC, tsh and cvi genes indicate that the isolate is avian pathogenic *E. coli* [42] Also, Timothy [43] reported that presence of these genes are associated with avian colibacillosis and indicates presence of APEC. Sixty-nine percent (186 *E. coli* isolates) of the current study considered as pAPEC according to [44] report that chicken *E. coli* isolates carrying > 5 VAGs were classified as pAPEC. Sit A and iuc genes both contributes to iron acquisition. Sit A is usually detected in APEC more than other commensal *E. coli* [42]. In this study sit A gene was detected with a high prevalence (97.3%) which is higher than the prevalence previously reported in Brazil, [45]. High prevalence of increased serum survival protein coded by *iss* gene (93.3%), was higher than what was detected in USA and Germany where 80.5 and 82.7% of APEC isolated from birds with colibacillosis possess such gene [46, 47]. *Tsh* genes were found in 46.4% of isolates, similar to the findings of Ewers et al. [47] and Dozois et al. [48] where *Tsh* genes were detected at a prevalence rate of 53.3 and 49.8%, respectively. Toxin-producing genes *astA was* detected in 71% of the isolates which is higher than the study of [49] were astA detected in 21% of the tested *E. coli*.

In general, VAGs are integrated within the plasmid, the pathogenicity islands (chromosomally or extra chromosomally) or the bacteriophages, the acquisition of VAGs is usually through horizontal gene transfer [50, 51] which may explain the absence or the low prevalence of the remaining VAGs.

### Antibiotic susceptibility

This study found lower resistance rates against beta lactams, tetracycline and fosfomycin than a previously reported [52]. However, a higher percentage of resistance was identified in isolates against enrofloxacin, spectinomycin, gentamicin and florfenicol [53].

In the present study, 93.3% of the APEC isolates were resistant to amoxicillin, which is lower than the resistance rate of 100% reported in Jordan by Abu-Basha et al., [52] and higher than the 83.3% resistance rate reported by Qabajah and Ashhab [53]. In this study, 5.1% of the isolates were resistant to aztreonam, which is significantly lower than the resistance rates (41.1%) previously reported by Ahmed et al. [34] in Eygpt. This lower rate of resistance is likely to be due to the fact azetronem is not used in poultry in Jordan. In this study, APEC isolates were found to be resistant to doxycycline (92.2%) and oxytetracycline (55%) which is lower than the 100% resistance rate reported by Abu-Basha et al., [52]. APEC isolates (57.2%) were found to be resistant to gentamycin, which is higher than previously reported [34, 52]. APEC isolates were found to be highly-resistant to spectinomycin (92.2%) compared to resistance rates (47%) previously reported by [52]. APEC isolate resistance to the cephalosporin’s; ceftazidime, ceftriaxone and cefepime showed the lowest resistance levels among the tested panel of antimicrobials this result is expected for these types of cephalosporins as they are not used in poultry industry.

Attention should be paid to those antimicrobials used in broilers feed, drinking water, and as growth promoter in suboptimal doses; chlortetracycline, erythromycin, enrofloxacin, oxytetracycline and sulfonamides. The high resistance levels observed for these antibiotic classes reflect the widespread use of them in poultry. In Jordan, high frequencies of antimicrobial resistance were found in chicken isolates that can be attributed to the large-scale use of antimicrobials for disease treatment and prevention without veterinary consultation.

### Antimicrobial Resistance genes

The current study targeted eight ARGs, commonly associated with antimicrobial resistance among APEC. For tetracycline resistance genes, TetA and TetB, 90.7% of the isolates expressed at least one of the tetracycline resistance genes, with TetA was the most prevalent gene. This is similar to a study carried out in Egypt, where 91.8% of APEC isolates from broilers, possessed tetracycline resistance genes, with the most prevalent type being TetB [34]. The high prevalence of tet genes are associated with high resistant against tetracycline class (resistance range from 55 to 92.2%). A high prevalence of the class 1 integron (int1) gene was expressed by 97% of the APEC isolates, which was higher than previously reported [39]. This finding highlighted the ability of the APEC isolates to capture ARG from other pathogenic bacteria and the environment. Sulphonamide resistance genes sul1 and sul2 were both prevalent in 70% of the APEC isolates, higher than a previous Portuguese study which found that APEC sul1 gene prevalence was 47% and sul2 was not tested [54]. Also, the relatively high prevalence of sul1 and 2 (70%) were associated with high resistant against Sulphamethoxazole (95.5%).

Genes encoding beta-lactamases; bla-_SHV_ and bla- _TEM_ was identified in the APEC isolates at a prevalence of 1.8 and 72.9%, respectively. This differs from the findings of Huijbers et al., [55] in the Netherlands who assessed the prevalence of ESBL producing *E. coli* in broiler and people living or working with broiler farms; Huijbers et al., [55] study reported much higher prevalence of bla-_SHV_ (17%) but lower bla- _TEM_ (9.1%). The prevalence of *Cat1* gene was 61.7% which is not significantly (*P* > 0.5) associated with high resistant to florfenicol (93.7%), this is may be due to presence of other Cat genes which are not tested.

Plasmids are considered as the main vector for horizontal gene transfer of ARGs. Increased levels of ARGs sulI, intI, aphA and traF in the aquatic environment facilitate the spread of AMR through plasmids. The high prevalence of integrons among APEC isolates (97%) which is reported to be responsible for the horizontal gene transfer and highly responsive to antimicrobial stress in the environment could explain the abundance of ARGs among the isolated APEC [56].

### Risk factors

This study correlates risk factors that were hypothesized to be associated with the presence of MDR *E. coli* in broiler farms in Jordan. The main risk factors associated with the presence of MDR *E. coli* were; farms using water from artesian wells, as poultry drinking water increases the incidence of having MDR *E. coli* compare to farms supplied by the municipalities’ drinking water. Jordan has 12 ground water basins that serve 282 million m^3^ of water. This water is used for both industrial and irrigation purposes [57] Water environments are considered as reservoirs and amplifying sources of antimicrobial resistant genes of clinical importance [58].

Previous studies, performed in Canada, tested the antimicrobial resistance of *Enterococcus* spp. Identify that 86, 58 and 100% of the isolates were resistant to more than one type of antibiotic in poultry litter, surface water and ground water isolates, respectively [59]. This finding suggests that there is a high presence of antibiotic resistant genes in surface water, wastewater, and poultry litter.

Furthermore, this study found that farms located in close proximity to other poultry farms were at high risk of contamination with MDR *E. coli* which is similar to finding of Hartung & Schukz [60], emphasized that serious pathogens are transmitted by air, which is positively correlated to farm density, considering farmers have no control over farm location. Therefore, farmers should pay attention toward wind directions in their area. Personal movement, vehicles and instruments can also be considered as vectors for transmission of pathogens.

Other potential risk factors related to antimicrobial usage were the use of antimicrobial agents as growth promoters and the administration of antibiotic without veterinary consultation. Many studies support that the improper use of antibiotics for increasing productivity, enhances the selection pressure for antimicrobial resistant pathogens [7, 61].

Public health concerns regarding antimicrobial residues and antimicrobial resistance pathogens in food and the environment reinforce the need for more research on safer alternatives to antibiotics as feed additives [19]. Netherlands was ranked as the highest antimicrobial consuming country in 2007, with an estimated 600 tons of therapeutic antimicrobials used in the veterinary sector. Therefore, the Netherlands set up a monitoring action plan to reduce the antimicrobial use in animals. The first step taken was to establish a veterinary medicine authority, whose main purpose was to record antimicrobial usage and prescription from farmers and Veterinarians, and to set species-specific annual targets for antimicrobial use. This action plan resulted in a 56% reduction in antimicrobial usage in the period between 2007 and 2012 [62].

## Conclusion

This study characterised the VAGs of avian pathogenic *E. coli* and establish their antimicrobial resistance patterns. The widespread of antimicrobial resistance of APEC isolates and detection of ARGs highlighted the need to monitor the spread of ARGs in poultry farms and the environment in Jordan. Use of ground water and closely located farms were significant risk factors associated with the presence of MDR APEC in broiler chickens in Jordan.

## Methods

### Sampling

#### Study area

Chicken samples were collected from farms located in northern Jordan; Irbid, Jerash, Ajlune, and Mafraq governorates, which contain 896 broiler farms with annual capacity 12, 064,600 bird [63].

### Sample size determination

According to the sample size formula from an infinite population:

$$ n=\kern0.5em \frac{z^2\  pq}{d^2} $$

Where; p = estimated prevalence of disease in the population, q = (1-p), d = accepted margin of error and Z the value for specific confidence level.

The confidence level is 95%, Z value = (1.96), Estimate prevalence = 88.2% [64], d = (0.05) thus, *n* = 159.8 farms.

Eighty-four farms were visited and asked to fill in the questionnaire before samples collection. Five hundred and four sick birds’ samples were collected during the period from April to December 2016.

#### Data collection

A questionnaire was designed with 42 questions divided into four sections, which covered the factors believed to be associated with antimicrobial resistance. The questionnaire was translated to Arabic and answered by the owners or the veterinarian of each farm during personal interviews while collecting the samples. The questionnaire was field pre-validated. (Additional file 1).

#### Isolation and conventional identification of APEC

Aseptic swabs from liver, heart, spleen and lungs of birds symptomatic of colibacillosis were cultured on 5% sheep blood agar and on MacConkey agar media (Oxoid), and subcultured on selective differential media eosin methylene blue agar (EMB) (Oxoid) [65]. The isolated bacteria were identified as *E. coli* by observing their cultural characteristics, morphology by Gram’s stain, oxidase test, biochemical reactions using indole, methyl-red, Voges-Preuskuar and citrate tests (IMViC), Kligler Iron Agar (KIA) and motility test as described by Tonu et al. [66]. The suspected isolates were maintained in cryostat tubes containing 20% glycerol with LB Luria Bertani broth at − 70 °C [26].

#### Confirmation of APEC using RapID™ ONE system

*E. coli* isolates were tested using RapID ONE system Kit (Remel, USA) as indicated in the kit catalogue, and results then were interpreted using ERIC (Remel RapID database).

#### APEC serotyping

Serotyping was conducted using *E. coli* polyvalent O antisera and mono-specific antisera prevalent in poultry; O1, O2, O78, O8, O9, O18, O26, O25, O45, O55, O86, O111, O114, O119, O127, and O128 [30, 31, 67]. All the *E. coli* isolates were subjected to serotyping according to the instructions of the manufacturer (SSI Diagnostica) using a micro titre plate agglutination test.

### Molecular identification of APEC

#### DNA extraction and detection of 16 s rRNA gene of *E. coli* by PCR

Extraction of DNA from the *Escherichia coli* was carried out by boiling procedure and rapid cooling method. In brief, a single colony of *E coli* was resuspended in 100 μl of nuclease free water and boiled for 10 min and immediately cooled on icebox followed by centrifugation at 10,000 rpm for 10 min. The supernatant was collected, stored at − 20 °C and used as DNA template [34].

*E. coli* isolates were confirmed by detection of 16_S_ rRNA gene using conventional PCR. As described by Hossain et al., [64]. Oligonucleotide primers sequences used for the amplification of 16S rRNA gene of *E. coli* was 16 s-F: GAC CTC GGT TTA GTT CAC AGA and 16 s-R: CAC ACG CTG ACG CTG ACC A, location within the gene 4,267,278–4,267,845 and amplicon size 485 bp. PCR reaction mixture consisted of 12.5 μl of 2 × PCR master mixtures (Promega), 10 pmol primer of each and 2 μl of genomic DNA in a final volume of 25 μl adjusted by nuclease free water. The cycling conditions consisted of initial denaturation at 95 °C for 5 min., followed by 30 cycles of 94 °C for 1 min., 55 °C for 45 s min. and 72 °C for 1 min., with final extension at 72 °C for 7 min. The amplified products were electrophoresed into 1.8% agarose gel at 100 V visualized under Gel doc/UV trans-illuminator.

#### Multiplex polymerase chain reaction method for detection of virulence associated genes (VAGs)

Each DNA extract was screened for 16 VAGs associated with avian pathogenic *E. coli*; *sfa*, *iss*, *tsh, kps, kpsII*, *kpsIII*, *iucC*, *iucD*, *hlyD*, *ibeA*, *sitA, astA, cvi, papC, irp2 and vat,* using a multiplex PCR [47]. Primers were obtained from GENEWIZ Company (USA) and Intron, South Korea supplied all PCR constituents used in this study. All sixteen primers sequences were given in [43]. Briefly, each 50 μl PCR reaction contained: 12 μl of 25 mM MgCl2, 21.3 μl nuclease free water, 5 μl 10x PCR buffer, 4 μl of 20 mM dNTPs, 0.3 μl of each 100 pmol forward and reverse primer, 0.3 μl, 5 U/ μl Taq polymerase and 5 μl template DNA. Thermocycler conditions were: initial denaturation 95 °C for 5 min; nine cycles of 95 °C for 60 s, 55 °C for 30 s, 72 °C for 60 s; twenty-eight cycles of 94 °C for 30 s, 55 °C for 30 s, 72 °C for 30 s with a final extension 72 °C for 7 min. The mixture was held at 4 °C. PCR products were subject to electrophoresis on a 2% agarose gel in tris–acetate buffer (TAE) at 150 V for 60 min alongside a super Ladder-Low 100 bp ladder (Intron, South Korea).

Two separate m-PCR assays were performed; one multiplex PCR previously described by Ewers et al. [47] and one m-PCR assays for ibeA and sitA described by Timothy et al. [43]. Briefly, for a 25 ml multiplex PCR, 4 μl of 25 mM MgCl2, 13.9 μl nuclease free water, 2.5 μl 10x PCR buffer, 0.5 μl 20 mM dNTPs, 0.1 μl of each 100 pmol forward and reverse primers, 0.5 μl 5 U/ μl Taq polymerase and 2 μl DNA templates were used. Multiplex PCR thermocycler conditions were as follows: initial denaturation 94 °C for 3 mints followed by 25 cycles of: 94 °C for 30 s, 58 °C for 30 s, 68 °C for 3 mints with a final extension 72 °C for 10 mints. The mixture was held at 4 °C. Each individual PCR contained 1 μl DNA template, 1 μl of each primer (100 pmol) and 22 μl of 1.1x Reddymix PCR master mix with 1.5 mM MgCl2. M-PCR thermocycler conditions for sitA and ibeA were; 95 °C for 12 min and 25 cycles of: 94 °C for 30 s, 63 °C for 30 s, 68 °C for 3 min; 72 °C for 10 min with a final hold 4 °C. PCR products were subject to electrophoresis as above. Isolates carrying > 5 VAGs were classified as APEC.

### Antimicrobial susceptibility

#### Standard disc diffusion method

The agar disk diffusion test was carried out according to [28]. All *E. coli* isolates were tested for 19 antibiotics: amoxicillin (25 μg), doxycycline (30 μg), ciprofloxacin (5 μg), ceftriaxone (30 μg), gentamicin (10 μg), florfenicol (30 μg), cefepime (30 μg), aztreonam (30 μg), imipenem (10 μg), cephalexin (30 μg), ceftazidime (30 μg), sulphamethoxazole-trimethoprim (23.75/1.25 μg), Amoxicillin-clavulanate (20/10 μg), apramycin (15 μg), spectinomycin (25 μg), Enrofloxacin (5 μg), Oxytetracycline (30 μg), Chlortetracycline (10 μg), and Fosfomycin (50 μg). *Escherichia coli* ATCC 25922 was used as control strain.

#### Minimal inhibitory concentration (MIC)

Susceptibility to 8 antimicrobials was evaluated by broth microdilution [28] Cationic-adjusted Muller-Hinton broth (Cationic-adjusted Muller-Hinton, Fluka, Switzerland) was used to prepare the bacterial inoculum and dilute the antimicrobial agents (Table 7). According to the MIC breakpoints, *E. coli* isolates that were resistant to 3 or more antimicrobial classes were considered multidrug-resistant isolates [44]. The reference *Escherichia coli* ATCC 25922 strain was used as a control strain.

**Table 7:** Antimicrobial agents used in the MIC with their potency and dilution solvent

Antibiotics	Potency(μg)	Dilution solvent (10 ml)	Wight (mg)
Gentamicin	618	Distilled water	323.62
Amoxicillin	998	Saturated NaHCO3	200.4
Ciprofloxacin	998	1 ml acetic acid+ 9 ml DW	200.2
Ceftazidime hydrate	983	Distilled water	203
Cephalexin	1000	1 M NH4OH	347.4
Ceftriaxone	1000	Distilled water	661.6
Florfenicol	990	Distilled water	479.0
Doxycycline	980	Distilled water	204.08

#### Molecular detection of antimicrobial resistant genes by multiplex PCR

PCR was conducted for the *E. coli* isolates that were found resistant to one or more of the previously mentioned antimicrobials, as described by [68]. The DNA templates from the DNA extraction step were used to detect resistance genes(Table 8).

**Table 8:** PCR target genes, primer sequence, PCR product size and annealing temperature

Target gene	Primer sequence	PCR product size (bp)	Annealing Temp.
tetA	tetAF GCT ACA TCC TGC TTG CCT TC	210	55
tetracycline	tetAR CAT AGA TCG CCG TGA AGA GG		
tetB	tetBF TTG GTT AGG GGC AAG TT1 T TG	659	55
tetracycline	tetBR GTA ATG GGC CAA TAA CAC CG		
blaTEM	TEMF ATT CTT GAA GAC GAA AGG GC	1150	60
beta lactams	TEMR A CG CTC AGT GGA ACG AAA AC		
blaSHV	SHVF CAC TCA AGG ATG TAT TGT G	885	60
beta lactams	SHVR TTA GCG TTG CCA GTG CTC G		
sul1 sulphonamide	sul1F CTT CGA TGA GAG CCG GCG GC	417	68
	sul1R GCA AGG CGG AAA CCC GCG CC		
sul2 sulphonamide	sul2F AGG GGG CAG ATG TGA TCG AC	249	58
	sul2R GCA GAT TTC GCC AAT TG		
Cat1 chloramphenicol	catF CCT GCC ACT CAT CGC AGT	623	55
	catR CCA CCG TTG ATA TAT CCC		
int1 integrons	int1F CCT CCC GCA CGA TGA TC	280	55
	int1R TCC ACG CAT CGT CAG GC		

### Statistical analysis

#### Data analysis

Eighty-four broiler farms completed the questionnaire and were included in the analysis using SPSS 21.0 software. Questions with the same answers were excluded from the analysis (application of “all in all out” strategy, disinfection of farm building before introduction of new flocks, application of vaccination program, previous history of respiratory diseases, monitoring of mortality rate and use of antimicrobials for disease treatment).

Chi-square (X^2^) and Fisher exact tests were performed to screen association between outcome variable (resistance status of the farm) and risk factors in univariable analysis. Only variables with *P* ≤ 0.25 considered for further analysis, which were used to perform the final logistic regression model. Collinearity between variables was tested using chi-square and Spearman rank correlation test in bivariate analysis.

#### Independent variable

The resistance status of a farm was used as unit of comparison, farms were categorized into resistance according to presence of one or more multidrug resistant APEC isolate coded as (1) and susceptible isolates coded as (0) depending on the multidrug resistance definition. According to WHO [69] five antimicrobial agents (OT, CN, CIP, AML and FOS) were selected in order to categorize the isolates into multidrug resistant patterns (resistant to three or more antimicrobials) and sensitive isolates [70].

#### Final multivariable logistic regression

Variables from univariate analysis step were used to perform multivariable logistic regression model for the outcome, risk factors were considered significant when *P* value ≤0.05, non-significant factors re-entered when a new variable become significant or removed. The final model was tested to fit hosmer and lemeshow-of-fit test.

## Additional files

Additional file 1: Questionnaire, Risk assessment of antibiotics resistance in broilers poultry farms In Jordan. (PDF 229 kb)

Additional file 2: Spearman correlation test. (PDF 115 kb)

## Abbreviations

AMR: Antimicrobial Resistance
APEC: Avian Pathogenic *E. coli*
ARG: Antimicrobial Resistance Genes
CRD: Chronic Respiratory Disease
DNA: Deoxyribonucleic acid
EB: Ethidium Bromide
EMB: Eosin Methylene Blue Agar
ExPEC: Extraintestinal Pathogenic *E. coli*
KIA: Kligler Iron Agar
LB: Luria Bertani Broth
MDR: Multidrug Resistant
MIC: Minimum Inhibitory Concentration
m-PCR: Multiplex Polymerase Chain Reaction
NMEC: Neonatal Meningitis *E. coli*
PCR: Polymerase Chain Reaction
QACs: Quaternary ammonium compounds
Rpm: Rounds per Minuet
rRNA: Ribosomal Ribonucleic Acid
TBE: Tris –Borate-EDTA
UK: United Kingdom
USA: United States of America
UV: Ultra violet
VAG: Virulence associated genes
