# Supplementary material for: Identification of Escherichia coli from broiler chickens in Jordan, their antimicrobial resistance, gene characterization and the associated risk factors
Source: BMC Vet Res. 2019 May 22;15:159. doi: 10.1186/s12917-019-1901-1 (PMC6530146; doi:10.1186/s12917-019-1901-1)
Supplement: Supplementary file 1 — Questionnaire, Risk assessment of antibiotics resistance in broilers poultry farms In Jordan. (PDF 229 kb) [file 12917_2019_1901_MOESM1_ESM.pdf]

Questionnaire

Risk Assignment of Antibiotics Resistance in Boilers Poultry Farms in Jordan

---

We're conducting research on the effect and relationship of farming practices on antimicrobials resistance on *E. coli*. The survey should only take 10 minutes. We would appreciate your time taken to complete this survey.

Farm owner: ---

Address: --

Phone number: ---

Farm capacity: ---

Date: ---

Part 1 : farm location and design

- In which geographical area your farm is located?

  □ valley □ plain □ mountain □ desert

- What is the direction of your poultry house?

  □ south to north □ east to west

- How far your farm is located from the high way and noise?

  □ on the high way □ far from the high way

- What is the type of ventilation system in your farm?

  □ natural □ mechanical

- Dose the farm apply "all in all out" policy?

  □ Yes □ No

- Is your farm composed of one or many houses

  □ One □ Many

- How far is your farm in relation to neighboring farms

  □ isolated □ very close

- Do wild birds have access to the poultry house?

  □ Yes □ No

- Are rodent present on your farm's facilities?

  □ Yes □ No

- Dose the farm have pest control protocol?

  □ Yes □ No

- How many times the trash (liter) is discarded from your farm?

□ One week □ two week's □ > one month □ none

Part 2: Water and feed supplement

- What is the present form of your farm feed material?

□ grounded □ pellets

- What is the water source used at your farm?

□ Municipalities water □ artesian well

- What is the type of water tanks?

□cement □ plastic □ metal

- How many times is the water tank cleaned?

□ monthly □ when needed □ between cycles □ none

- How many times is the water tanks disinfected?

□ monthly □ when needed □ between cycles □ none

- Type/s of disinfectant used.

□ one type □ mixed types

Part 3: Biosecurity and management

- Is the farm building disinfected before introduce the new chicken flock?

  □ Yes □ No

- Do you clean and disinfect feeders and drinkers before introduce the new chicken flock?

  □ Yes □ No

- Do you use disinfectant at the farm entrance to disinfect visitors' boots and vehicles tires before entering the farm?

  □ Yes □ No

- How many times do you disinfect your poultry houses?

  □ during the cycle □ between cycles □ when needed □ never

- Do the workers wear protective clothes when handling the birds?

  □ Yes □ No

- Does the workers change or disinfect their outfit when they work between different houses?

  □ Yes □ No

- Is the farm entrance is restricted for unauthorized traffic?

  □ Yes □ No

- Number of people working in the farm.

  □ one worker □ more than one worker

Part 4: Diseases and antimicrobial used

- What are the most common diseases in your farm?

  □ Yes □ No

- Dose the farm keep records of daily mortality?

  □ Yes □ No

- Dose the farm apply a specific vaccination program?

  □ Yes □ No

- What do you use antibiotics for?

  □ Disease treatment □ Disease prevention □ Growth promoter

- If you use antibiotics for growth as promoter, please list the main ones that you use:

- If you use antibiotics for disease prevention, please list the main ones that you use:

- Do you get a prescription from your veterinarian before getting antibiotics for your flock?

□ Yes □ No

- Does your veterinarian see your sick chickens and do necropsy before prescribing antibiotics?

  □ Yes □ No

- If you treat your animals yourself, where do you get information on how to do so?

  □ Veterinarian □ Feed store □ Friends/neighbors □ Training □ Other

- Do you keep antimicrobials in your farm?

  □ Yes □ No

- When a treatment doesn't work, what do you do?

  □ Increase the dose □ Change drugs □ Increase the treatment period

- When you buy antibiotics, what factors do you take into account?

  □ Price □ Brand □ Packaging

  □ Quality □ Easy to obtain

  □ Recommendation of the vet/vendor □ Previous experience

- Frequency of antibiotic use during the growing period?

- Do you perform antibiotic sensitivity testing before giving treatment to your flock?

□ Yes □ No

- How often does the veterinarian visit your farm?

- Do you know what drug withdrawal/withholding times are?

□ Yes □ No

- Have you ever observed any complications/side effects after administering antibiotics?

□ Yes □ No
