# Supplementary material for: Identification of Escherichia coli from broiler chickens in Jordan, their antimicrobial resistance, gene characterization and the associated risk factors
Source: BMC Vet Res. 2019 May 22;15:159. doi: 10.1186/s12917-019-1901-1 (PMC6530146; doi:10.1186/s12917-019-1901-1)
Supplement: Supplementary file 2 — Spearman correlation test. (PDF 115 kb) [file 12917_2019_1901_MOESM2_ESM.pdf]

Spearman Correlations Test

			poultry house direction	distance to the high way and noise	distance in relation to other farms	water source	workers wear protective cloths when handling the birds	farm entrance is restricted	use antibiotic for disease prevention	0 /1 or2 Information source	0 or 1 /2 Information source	0 or 2 / 1 Information source	use antibiotic as growth promoters	get prescription before use antibiotics	type of feed material used
Spearman's rho	poultry house direction	Correlation Coefficient	1.000	-.467-**	.277*	.573**	-.372-**	-.410-**	.281**	.211	.247*	-.065-	.288**	-.211-	.023
		Sig. (2-tailed)	.	.000	.011	.000	.000	.000	.009	.054	.023	.554	.008	.054	.835
		N	84	84	84	84	84	84	84	84	84	84	84	84	84
	distance to the high way and noise	Correlation Coefficient	-.467-**	1.000	-.306-**	-.251-*	.288**	.251*	-.209-	-.210-	-.185-	-.062-	-.172-	-.066-	-.115-
		Sig. (2-tailed)	.000	.	.005	.022	.008	.021	.056	.055	.093	.573	.117	.552	.298
		N	84	84	84	84	84	84	84	84	84	84	84	84	84
	distance in relation to other farms	Correlation Coefficient	.277*	-.306-**	1.000	.151	-.396-**	-.132-	.129	.202	.262*	-.113-	.090	-.087-	.296**
		Sig. (2-tailed)	.011	.005	.	.171	.000	.230	.242	.065	.016	.304	.415	.429	.006
		N	84	84	84	84	84	84	84	84	84	84	84	84	84
	water source	Correlation Coefficient	.573**	-.251-*	.151	1.000	-.169-	-.302-**	.180	.180	.252*	-.141-	-.073-	-.200-	.067
		Sig. (2-tailed)	.000	.022	.171	.	.125	.005	.102	.102	.021	.202	.510	.069	.542
		N	84	84	84	84	84	84	84	84	84	84	84	84	84
	workers wear protective cloths when handling the birds	Correlation Coefficient	-.372-**	.288**	-.396-**	-.169-	1.000	.153	-.144-	-.078-	-.014-	-.136-	-.131-	.175	-.043-
		Sig. (2-tailed)	.000	.008	.000	.125	.	.166	.193	.478	.900	.217	.236	.110	.698
		N	84	84	84	84	84	84	84	84	84	84	84	84	84
	farm entrance is restricted	Correlation Coefficient	-.410-**	.251*	-.132-	-.302-**	.153	1.000	-.262-*	-.217-*	-.207-	-.031-	-.194-	-.027-	-.010-
		Sig. (2-tailed)	.000	.021	.230	.005	.166	.	.016	.047	.059	.776	.077	.810	.929
		N	84	84	84	84	84	84	84	84	84	84	84	84	84
	use antibiotic for disease prevention	Correlation Coefficient	.281**	-.209-	.129	.180	-.144-	-.262-*	1.000	.465**	.473**	.004	-.041-	-.237-*	.066
		Sig. (2-tailed)	.009	.056	.242	.102	.193	.016	.	.000	.000	.974	.710	.030	.550
		N	84	84	84	84	84	84	84	84	84	84	84	84	84
	0 /1 or2	Correlation Coefficient	.211	-.210-	.202	.180	-.078-	-.217-*	.465**	1.000	.884**	.284**	.031	-.284-**	.007
		Sig. (2-tailed)	.054	.055	.065	.102	.478	.047	.000	.	.000	.009	.783	.009	.948
		N	84	84	84	84	84	84	84	84	84	84	84	84	84
	0 or 1 /2	Correlation Coefficient	.247*	-.185-	.262*	.252*	-.014-	-.207-	.473**	.884**	1.000	-.197-	-.029-	-.330-**	.059
		Sig. (2-tailed)	.023	.093	.016	.021	.900	.059	.000	.000	.	.072	.793	.002	.593
		N	84	84	84	84	84	84	84	84	84	84	84	84	84
	0 or 2 / 1	Correlation Coefficient	-.065-	-.062-	-.113-	-.141-	-.136-	-.031-	.004	.284**	-.197-	1.000	.124	.082	-.106-
		Sig. (2-tailed)	.554	.573	.304	.202	.217	.776	.974	.009	.072	.	.262	.460	.336
		N	84	84	84	84	84	84	84	84	84	84	84	84	84

	use antibiotic as growth promoters	Correlation Coefficient	.288**	-.172-	.090	-.073-	-.131-	-.194-	-.041-	.031	-.029-	.124	1.000	.062	-.063-
		Sig. (2-tailed)	.008	.117	.415	.510	.236	.077	.710	.783	.793	.262	.	.572	.572
		N	84	84	84	84	84	84	84	84	84	84	84	84	84
	get prescription before use antibiotics	Correlation Coefficient	-.211-	-.066-	-.087-	-.200-	.175	-.027-	-.237-*	-.284-**	-.330-**	.082	.062	1.000	-.098-
		Sig. (2-tailed)	.054	.552	.429	.069	.110	.810	.030	.009	.002	.460	.572	.	.376
		N	84	84	84	84	84	84	84	84	84	84	84	84	84
	type of feed material used	Correlation Coefficient	.023	-.115-	.296**	.067	-.043-	-.010-	.066	.007	.059	-.106-	-.063-	-.098-	1.000
		Sig. (2-tailed)	.835	.298	.006	.542	.698	.929	.550	.948	.593	.336	.572	.376	.
		N	84	84	84	84	84	84	84	84	84	84	84	84	84

**. Correlation is significant at the 0.01 level (2-tailed).

*. Correlation is significant at the 0.05 level (2-tailed).
